# Association between Milk Intake and Incident Stroke among Japanese Community Dwellers: The Iwate-KENCO Study

**DOI:** 10.3390/nu13113781

**Published:** 2021-10-25

**Authors:** Kozo Tanno, Yuki Yonekura, Nagako Okuda, Toru Kuribayashi, En Yabe, Megumi Tsubota-Utsugi, Shinichi Omama, Toshiyuki Onoda, Masaki Ohsawa, Kuniaki Ogasawara, Fumitaka Tanaka, Koichi Asahi, Ryo Itabashi, Shigeki Ito, Yasushi Ishigaki, Fumiaki Takahashi, Makoto Koshiyama, Ryohei Sasaki, Daisuke Fujimaki, Nobuyuki Takanashi, Eri Takusari, Kiyomi Sakata, Akira Okayama

**Affiliations:** 1 Department of Hygiene and Preventive Medicine, Iwate Medical University, Yahaba 028-3694, Japan; mutsugky@iwate-med.ac.jp (M.T.-U.); m02075df@jichi.ac.jp (D.F.); pears_takanashi@ybb.ne.jp (N.T.); ertakus@iwate-med.ac.jp (E.T.); ksakata@iwate-med.ac.jp (K.S.); 2 Department of Nursing Informatics, Graduate School of Nursing Science, St. Luke’s International University, Tokyo 104-0044, Japan; yyonekura@slcn.ac.jp; 3 Department of Health Science, Kyoto Prefectural University, Kyoto 606-8522, Japan; nokuda@kpu.ac.jp; 4 Faculty of Humanities and Social Sciences, Iwate University, Morioka 020-8550, Japan; kuri@iwate-u.ac.jp; 5 Department of Health Food Sciences, University of Human Arts and Sciences, Saitama 399-8539, Japan; en_yabe@human.ac.jp; 6 Iwate Prefectural Advanced Critical Care and Emergency Center, Iwate Medical University, Yahaba 028-3694, Japan; somama@iwate-med.ac.jp; 7 Health Care Center, Iwate University, Morioka 020-8550, Japan; onodat@iwate-u.ac.jp; 8 Department of Internal Medicine, Morioka Tsunagi Onsen Hospital, Morioka 020-0055, Japan; m-ohsawa@k2.dion.ne.jp; 9 Department of Neurosurgery, Iwate Medical University, Yahaba 028-3695, Japan; kuogasa@iwate-med.ac.jp; 10 Division of Nephrology and Hypertension, Department of Internal Medicine, Iwate Medical University, Yahaba 028-3695, Japan; ftanaka@iwate-med.ac.jp (F.T.); asahik@iwate-med.ac.jp (K.A.); 11 Stroke Center, Division of Neurology and Gerontology, Department of Internal Medicine, Iwate Medical University, Yahaba 028-3695, Japan; ritabash@iwate-med.ac.jp; 12 Division of Hematology and Oncology, Department of Internal Medicine, Iwate Medical University, Yahaba 028-3695, Japan; shigei@iwate-med.ac.jp; 13 Division of Diabetes, Metabolism and Endocrinology, Department of Internal Medicine, Iwate Medical University, Yahaba 028-3695, Japan; ishigaki@iwate-med.ac.jp; 14 Department of Information Science, Iwate Medical University, Yahaba 028-3694, Japan; ftakahas@iwate-med.ac.jp; 15 Iwate Health Service Association, Morioka 020-8585, Japan; koshi-m@aogiri.org; 16 Center for Liberal Arts and Sciences, Department of Human Sciences, Iwate Medical University, Yahaba 028-3694, Japan; ryou-hei-1115@topaz.ocn.ne.jp; 17 The Research Institute of Strategy for Prevention, Tokyo 103-0006, Japan; aokayama@jrisp.com

**Keywords:** milk intake, stroke, prospective cohort study, Japanese population

## Abstract

We aimed to evaluate the association between the milk consumption and incident stroke in a Japanese population, where milk consumption is lower than that of Western countries. In total, 14,121 participants (4253 men and 9868 women) aged 40–69 years, free from cardiovascular diseases (CVD) were prospectively followed for 10.7 years. Participants were categorized into four groups according to the milk intake frequency obtained from a brief-type self-administered diet questionnaire. The adjusted HRs of total stroke, ischemic stroke and haemorrhagic stroke associated with milk intake frequency were calculated using the Cox proportional hazards model. During the follow-up, 478 stroke cases were detected (208 men and 270 women). Compared to women with a milk intake of <2 cups/week, those with an intake of 7 to <12 cups/week had a significantly low risk of ischemic stroke in a model adjusting CVD risk factors; the HR (95% CI) was 0.53 (0.32–0.88). No significant associations were found in men. This study suggested that milk intake of 7 to <12 cups/week decreased the risk of ischemic stroke in Japanese women. Milk intake of about 1 to <2 cups/day may be effective in the primary prevention of ischemic stroke in a population with low milk intake.

## 1. Introduction

Milk and dairy products are the major components of traditional Western diets, and the effects of their consumption on health have been frequently reported [1]. In Japan, milk intake is a relatively new dietary habit that was introduced to Japan after World War II, when it was served in school lunches. According to the 2017 Food Agriculture Organization balance sheet, the food supply quantity of milk was 245.43 kg/capita/year and 215.96 kg/capita/year in North America and Europe, respectively, compared to 58.63 kg/capita/year in Japan, corresponding to 160 g/capita/day [2]. The consumption of milk in Japan is lower than that in Western countries, and it is much lower for adults. According to the National Health and Nutrition Survey conducted in Japan in 2016, the average milk and dairy consumption was 111.2 g/day for those aged 20 years and above and 306 g/day for those aged 7–14 years [3]. Conversely, according to National Health and Nutrition Survey in US in 2007–2010, the average total dairy consumption was 2.1 cup equivalents/day for those aged 9–18 years, 1.7 cup equivalents/day for those aged 19–50 years, and 1.5 cup equivalent/day for those aged 51–70 years [4]. The Dutch National Food Consumption Survey 2012–2016 showed that the mean dairy product consumption was 374 g/day for men aged 19–70 years and 321 g/day for women aged 19–70 years [5].

The association between milk consumption and the risk of cardiovascular diseases (CVDs), such as myocardial infarction and stroke, has also been often reported [6,7,8]. The epidemiology of the diseases differs among Japanese and Western populations; in Western countries, mortality and morbidity from coronary artery disease are higher than those from stroke, while in East Asian countries, including Japan, mortality and morbidity from stroke are higher than those from myocardial infarction [9]. The reports on health effects of milk consumption that focus mainly on Western populations may not provide sufficient evidence for the Japanese population, which differs greatly from the Western population in terms of both milk intake and disease patterns.

Milk is a good source of potassium, calcium, and magnesium, which have been reported to reduce blood pressure [10,11,12]. In observational and interventional studies, an inverse relationship of milk consumption with blood pressure levels, and the development of hypertension have been observed [13,14]. Therefore, milk consumption is expected to have a potential risk-reducing effect on stroke.

A recent meta-analysis showed a significant inverse association between milk consumption and the risk of stroke [15,16], while others have shown a null association [17]. Some meta-analyses have suggested a nonlinear relationship [18,19]. Several studies have examined the association of milk consumption with mortality from stroke in Japanese people, but the results have been inconsistent [20,21,22]. In addition, to the best of our knowledge, no study involving the Japanese subjects has used the incidence of stroke as an endpoint. Therefore, the relationship between milk consumption and the incidence of stroke in the Japanese population is unclear.

Therefore, this study aimed to elucidate the relationship between the frequency of milk intake and the incidence of stroke in a Japanese population, whose milk consumption is lower than that in Western populations.

## 2. Materials and Methods

### 2.1. Study Population

**Figure 1:**
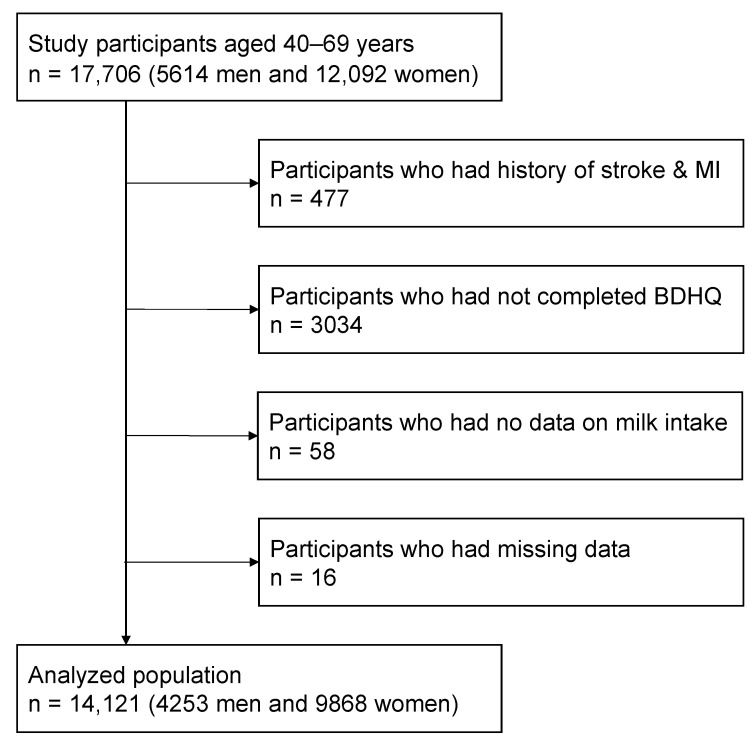
Flowchart for the selection of analyzed cohort.

The Iwate-Kenpoku cohort (Iwate-KENCO) study is a prospective cohort study of community-dwelling residents in the Ninohe, Kuji, and Miyako districts of the northern part of Iwate Prefecture, Northeast of the main island of Japan. The methodology of the Iwate-KENCO study has been described elsewhere [23]. A baseline survey was conducted between 2002 and 2005, wherein participants were recruited from the individuals who participated in the government-regulated multiphasic health check-up in each municipality. In total, 17,706 participants (5614 men and 12,092 women) aged 40 to 69 years provided written informed consent for participation in this study. In the present analysis, we excluded 3585 persons for the following reasons: 477 persons with a history of stroke or myocardial infarction, 3092 persons without the data for food frequency questionnaire, and 16 persons with missing data. Consequently, data from 14,121 participants (4253 men and 9868 women) were analyzed in this study (Figure 1). 

### 2.2. Frequency of Milk Intake and Other Foods

Food intake was assessed once at baseline using the brief-type self-administered diet history questionnaire (BDHQ) [24,25], which includes questions about the frequency of consumption and/or portion sizes of about 58 food items. The BDHQ was validated through comparison with results from dietary records, and the Spearman’s correlation coefficient for dairy products was 0.70, which indicated reasonable ranking ability [25]. Two questions regarding dairy foods intake were posed to assess the consumption (number of cups) of normal/high-fat milk/yogurt and low-fat milk/yogurt. Frequency in the BDHQ were measured as “≥2 cups/day”, “1 cup/day”, “4 to 6 cups/week”, “2 to 3 cups/week”, “1 cup/week”, “<1 cup/day”, and “never drink”. These were re-categorized as 14, 7, 5, 2.5, 1, 0.5, and 0 cups/week, respectively, in the present analysis. We calculated the total number of cups of normal/high-fat and low-fat milk/yogurt and described it as milk consumption (cups/week). Based on milk intake, participants were categorized into following four groups: <2 cups/week, 2 to <7 cups/week, 7 to <12 cups/week, and ≥12 cups/week. 

To examine the food intake pattern, we calculated the intake frequency (times/week) of starchy foods (such as rice, bread, and noodles), fish, soy products, meat, vegetables, fruits, and sugar-sweetened beverages, and the number of cups of miso soup per day. The frequency ratio of the total fish and soy products intake to that of meat (times/times) was also calculated.

### 2.3. Stroke Event Ascertainment

Stroke events were identified by accessing the Iwate Stroke Registry, which included the entire area where the participants lived; indeed, details of this registry have been described previously [26,27]. Since 1991, the stroke registration program has been coordinated by the Iwate Prefecture government and the Iwate Medical Association; the medical records of all medical facilities within the survey area are verified to ensure complete capture of all data. The stroke diagnostic criteria in this registry are based principally on the criteria established for the Monitoring System for Cardiovascular Disease commissioned by the Ministry of Health and Welfare [28], and these criteria correspond with those published by the World Health Organization [29]. To verify the accuracy of the data, a physician or trained research nurse visited and checked the medical records at the referral hospitals. We defined the follow-up period as the period from the baseline survey to either the first outcome or the end of the observation period. In the present study, we used follow-up data until 31 December 2014; participants who did not experience any outcomes during the follow-up period and those who moved out of the study area were censored administratively. Death and date of death were confirmed by the investigators reviewing the population-register sheets of the cohort members.

### 2.4. Other Measurements

The baseline survey consisted of a self-reported questionnaire, measurements of anthropometric data and blood pressure, and blood tests. The methodology for data collection has been described previously [23]. Body mass index (BMI) was calculated by measuring height and body weight. BMI was classified into four categories: <18.5 kg/m^2^, 18.5–24.9 kg/m^2^, 25–29.9 kg/m^2^, and ≥30 kg/m^2^. Systolic blood pressure (SBP) and diastolic blood pressure were recorded two times after five minutes of sitting rest, and the mean of the two measurements was used. Casual blood samples were drawn from the antecubital vein. Glycated haemoglobin (HbA1c) levels were measured using high-performance liquid chromatography. Serum total cholesterol (TC) and high-density lipoprotein cholesterol (HDLC) levels were measured by direct enzymatic assays.

Participants completed a self-reported questionnaire and reported their use of antihypertensives, smoking status, alcohol consumption status, and exercise habits. We asked participants to complete the questionnaire and bring it to their municipal health check-up site. In cases where the answers in the questionnaire were insufficient, a trained interviewer asked the respondent to answer as fully as possible. Smoking status was categorized into three groups: current smoking, ex-smoking, and non-smoking. Alcohol consumption was assessed by the frequency per week and amount of drinking per occasion and categorized as follows: intake of ≥3 drinks/day, 2 to <3 drinks/day, <2 drinks/day, ex-drinking, and non-drinking for men. Women reported less frequent alcohol intake compared with men, and the ≥3 drinks/day and 2 to <3 drinks/day categories were aggregated. Regular exercise was defined as engaging in exercise for at least 60 minutes, 8 times per month. In women, menopausal state was divided into two categories based on the answer to a questionnaire: postmenopausal state or not.

### 2.5. Statistical Analysis

All analyses were stratified by sex. Linear trends across the four milk intake frequency categories were estimated by one-way analysis of variance (ANOVA) for continuous variables and chi-square test for categorical variables. Using the Cox proportional hazards model, multivariate adjusted hazard ratios (HRs) and 95% confidence intervals (CIs) for total stroke, ischemic stroke, and haemorrhagic stroke in each group, considering the category “<2 cups/week” as a reference, were calculated for 4 models: model 1, adjusted for age; model 2, adjusted for age and lifestyle factors (smoking status, alcohol consumption status, and exercise habits); model 3, adjusted for age, lifestyle factors, and dietary factors (fruits and vegetables, fish and soy products intake to meat intake ratio); and model 4, adjusted for age, lifestyle factors, dietary factors, BMI categories, menopausal state, and CVD risk factors (SBP, HbA1c, TC, HDLC, and use of antihypertensives). The assumption of proportional hazard was verified using an interaction term between time and milk intake frequency in the models. All *p* values were two-tailed, and differences with *p* values < 0.05 were considered statistically significant. Statistical analyses were performed using the SPSS software package, version 25 (IBM Corporation, Armonk, NY, USA).

## 3. Results

The numbers (percentages) of men in the milk intake frequency categories were 1072 (25.2%), 1129 (26.5%), 1508 (35.5%), and 544 (12.8%) for <2 cups/week, 2 to <7 cups/week, 7 to <12 cups/week, and ≥12 cups/week, respectively. The corresponding numbers (percentages) for women were 1370 (13.9%), 2624 (26.6%), 4257 (43.1%), and 1617 (16.4%), respectively. Table 1 shows the baseline characteristics of the participants according to milk intake frequency categories. The men who had a higher milk intake frequency were older, had lower SBP, and significantly increased TC and HDLC levels; additionally, the proportion of current smokers and those who consumed ≥3 alcoholic drinks/day was significantly lower, and the proportion of those with regular exercise habit was significantly higher. Similar trends were observed among women; additionally, the proportion of obese women was significantly lower, and the proportion of postmenopausal women was significantly higher among those with a higher milk intake frequency.

Table 2 shows the food intake frequency of the participants according to milk intake frequency categories. The intake of starchy foods was similar among the groups. Participants with a higher milk intake frequency consumed more vegetables, fruits, meat, fish, and soy products, and the ratio of total fish and soy products intake to meat intake was significantly higher for those with a higher milk intake frequency.

The total observational person-years was 152,518 and the mean (standard deviation) observational period was 10.7 (2.1) years. Total stroke, ischemic stroke, and haemorrhagic stroke occurred in 478 (208 men and 270 women), 263 (134 men and 129 women), and 210 (72 men and 138 women) participants, respectively. Table 3 shows the multivariate adjusted HRs (95% CIs) of groups based on milk intake frequency, while considering the <2 cups/week category as a reference. For total stroke, women consuming 2 to <7 cups/week and 7 to <12 cups/week had significantly lower risks in models 1, 2, and 3. The HR was attenuated in model 4 with a marginal significance; the HRs (95% CIs) were 0.71 (0.47–1.05) (*p* = 0.084) and 0.73 (0.51–1.05) (*p* = 0.084) for the 2 to <7 cups/week and 7 to <12 cups/week categories, respectively. For ischemic stroke, women who consumed 2 to <7 cups/week had a significantly lower risk in model 1, but the significance disappeared in further adjusted models. For women who consumed 7 to <12 cups/week, a significantly lower ischemic stroke risk was shown in all models; the HR was 0.53 (0.32–0.88) (*p* = 0.014) in model 4. For haemorrhagic stroke, no significant association with milk intake frequency was observed. In men, no significant associations were found between milk intake frequency and the risks of total stroke, ischemic stroke, and haemorrhagic stroke. Additionally, no linear relationships were observed in all models in both sexes.

## 4. Discussion

We demonstrated that women who consumed milk at the frequency of 2 to <7 cups/week and 7 to <12 cups/week tended to have a decreased risk of total stroke compared with those who consumed <2 cups/week; however, the statistical significance disappeared after adjusting for not only CVD risk factors such as BMI, SBP, TC and HPLC, but also lifestyle factors, dietary factors, and menopausal state. For ischemic stroke, the reduced risk for women who consumed 7 to <12 cups/week remained significant even after fully adjusting. In contrast, no significant association between the frequency of milk intake and incidence of stroke was observed among men.

The findings of the previous studies on the association between milk intake frequency and risk of stroke in Japanese cohorts have been inconsistent [20,21,22]. In a 15-year follow-up cohort study that was conducted in 1965, people who consumed milk ≥4 times/week had significantly lower mortality risks from total, haemorrhagic, and ischemic stroke than those who consumed milk <once/week [20]. In contrast, in a 16-year follow-up study that was conducted in 1979, no significant association between milk consumption and mortality due to stroke was observed [21]. NIPPON DATA80, a 24-year follow-up study that was conducted in 1980, showed an inverse relationship between milk and dairy consumption and mortality risk due to stroke with a marginal significance only in women [22]. These studies examined only the risk of mortality but not the incidence of stroke. To the best of our knowledge, this is the first prospective cohort study in Japan to investigate the association between milk intake frequency and the incidence of stroke.

Several studies regarding the association between milk consumption and the risk of stroke have found an inverse [30,31], non-significant [32,33,34], or even a positive association in Western populations [35]. A recent meta-analysis showed that the relative risk of stroke (incidence and mortality) for a 200 g increase in daily milk intake was 0.98 (95% CI, 0.95–1.01) in Western countries and 0.82 (95% CI, 0.75–0.90) in East Asian countries, including Japan [18]. The analysis also suggested a possible nonlinear relationship between milk intake and the risk of stroke, and the greatest reduction in the risk of stroke was observed with the intake of approximately 125 g of milk/day for Western populations. Regional differences were also suggested, and the greatest reduction in the risk of stroke was observed with the intake of approximately 165 g of milk/day for East Asian countries [18]. The findings of our study suggested that, compared to those with a milk intake of <2 cups/week, the HR for ischemic stroke in women was significantly lowest for those who consumed 7 to <12 cups/week, while the HR was not significant for those who consumed ≥12 cups/week. Our results also suggested a nonlinear relationship between milk intake frequency and stroke incidence. In the BDHQ, participants were asked to state the number of cups of milk without specifying the volume of the cup. However, people often use a 150–200 mL-cup or glass in Japan [36], and the current analysis may suggest the optimal amount of milk intake for the prevention of ischemic stroke in the Japanese population.

The present study could not elucidate the underlying mechanism for the association between milk intake frequency and stroke risk reduction; however, there are several possible explanations. First, people with higher milk consumption seemed to prefer traditional Japanese foods; the consumption of high amounts of fish and soy products was accompanied by higher vegetable and fruit consumption. These foods have been reported to be associated with the lower risk of mortality due to CVDs [37,38,39]. Current findings suggesting a favorable association between milk intake frequency and the risk of stroke may be partially affected by the preference for Japanese foods. Second, nutrients included in milk might have played an important role in reducing the risk of stroke; indeed, milk contains an abundance of minerals such as potassium, calcium, and magnesium. These minerals have been reported to be associated with the reduced risk of stroke [40,41,42]. A prospective cohort study has shown a decrease in the risk of stroke and ischemic stroke after a higher consumption of dairy-derived calcium [43]. It has also been suggested that the proteins and peptides in milk may have antihypertensive and insulin secretion control effects [44]. An inverse association has been reported between milk and dairy consumption and CVD risk factors, such as hypertension, diabetes, and metabolic syndrome [13,45,46]. In the present analysis, the significant association of milk intake frequency with stroke risk reduction weakened or disappeared after adjusting for CVD risk factors. High blood pressure levels and impaired glucose tolerance may mediate the association between milk intake frequency and the incidence of stroke. In contrast, as milk intake increases, the intake of saturated fat, an important determinant of blood cholesterol levels, of which the average intake of Japanese adults is 15.3 g/day [3], increases. However, a recent meta-analysis reported an inverse association between dietary saturated fat intake and stroke risk in Japanese, but not in non-Japanese [47]. Yamagishi et al. suggest that an increase in saturated fat intake to a level of about 20 g/day may be optimal for the primary prevention of CVD in the Japanese population [48].

In the present study, no significant association was observed between milk intake frequency and the incidence of stroke in men. Among men, the proportion of those who consumed 7 to <12 cups of milk/week was lower than that among women. Owing to a smaller number of men who consumed the quantity of milk that was effective in reducing stroke risk, there might not have been a significant association. Moreover, a higher proportion of men had CVD risk factors, such as hypertension, diabetes, heavy drinking, and smoking habit, compared to women. These factors might have had a stronger impact on the incidence of stroke than the milk consumption, which might have masked the association between milk intake frequency and the incidence of stroke.

Our study has several limitations. First, in the BDHQ, consumption of milk and yogurt was combined in one question, and these could not be distinguished. Additionally, we could not distinguish between the effects of normal- and low-fat milk because approximately 30% of the participants reported drinking both types of milk. However, in the baseline year (2003) of the present cohort study, most milk and dairy products consumed were in the form of whole milk (76.1%), according to the National Health and Nutrition Survey in Japan [49]. In addition, according to the 2003 statistical survey on milk and dairy products, the amount of milk produced in Iwate Prefecture was 97,530 kL, while the amount of fermented milk produced was 4951 kL [50]. In 2004, the purchase volume per week per household for whole milk and low-fat milk was 2.56 L and 0.43 L, respectively [51]. Therefore, we believe that normal- and low-fat milk and yogurt consumption can be considered as whole milk consumption in our cohort. The consumption of low-fat milk is increasing in Japan, and the impact of different types of milk requires investigation in the future. Second, the BDHQ contained the question only on the number of cups of milk without specifying the volume, which might have caused a misclassification of the milk consumption categories. However, given the nature of food frequency questionnaires, a quantitative assessment of nutrient intakes could not be expected. Only frequencies were assessed for most of the foods in the BDHQ, and the validation paper showed the BDHQ’s limited ability to estimate the mean values of nutrients [52]. Although we also did not consider total energy intake in this study, considering the substantial correlation coefficient (0.70) of the BDHQ, we believe we categorized the participants reasonably: participants with higher milk/yogurt consumption and lower consumption. Third, the generalizability of our findings may be limited. The participants were those who underwent health check-up in three districts in one prefecture and might have been highly health conscious. Therefore, they are more likely to have favorable health behaviors, and the incidence and hazard ratios of stroke may be underestimated. In addition, it may be difficult to extrapolate our findings to the Western populations because of the differences in milk consumption levels. However, we believe that our findings may be generalizable to the East Asian population, which share some common characteristics such as low milk intake, high salt intake [53], and relatively low levels of obesity [54] with Japanese populations.

In conclusion, our findings suggest that moderate milk consumption (7 to <12 cups/week) decreased the risk of ischemic stroke in women but not in men. Consuming approximately 1 to <2 cups of milk/day may be effective in preventing ischemic stroke in the Japanese population.

## Figures and Tables

**Table 1:** Baseline characteristics of participants according to milk intake frequency categories by sex, 2002–2005, Iwate-KENCO study.

Milk Intake Frequency		<2 Cups/Week	2 to <7 Cups/Week	7 to <12 Cups/Week	≥12 Cups/week	*p* for Trend
Men						
Number of participants		1072	1129	1508	544	
Age (years)		56.7 (8.6)	57.9 (8.1)	60.4 (7.1)	60.1 (7.5)	<0.001
SBP (mmHg)		128.9 (19.0)	129.9 (19.2)	128.3 (19.1)	127.5 (19.3)	0.050
HbA1c (%)		5.06 (0.66)	5.12 (0.78)	5.16 (0.74)	5.11 (0.72)	0.077
TC (mg/dL)		191.2 (33.5)	192.7 (33.1)	195.6 (31.8)	199.8 (32.6)	<0.001
HDLC (mg/dL)		56.6 (15.4)	55.2 (15.0)	56.3 (15.5)	58.8 (15.8)	0.003
BMI	<18.5 kg/m^2^	18 (1.7)	14 (1.2)	28 (1.9)	4 (0.7)	0.532
	18.5 to <25 kg/m^2^	687 (64.1)	667 (59.1)	935 (62.0)	333 (61.2)	0.418
	25 to <30 kg/m^2^	329 (30.7)	407 (36.0)	501 (33.2)	190 (34.9)	0.185
	≥30 kg/m^2^	38 (3.5)	41 (3.6)	44 (2.9)	17 (3.1)	0.380
Smoking status	Current smoking	519 (48.4)	446 (39.5)	436 (28.9)	143 (26.3)	<0.001
	Ex-smoking	252 (23.5)	315 (27.9)	457 (30.3)	170 (31.3)	<0.001
	Non-smoking	301 (28.1)	368 (32.6)	615 (40.8)	231 (42.5)	<0.001
Alcohol drinking status	≥3 drinks/day	113 (10.5)	80 (7.1)	61 (4.0)	27 (5.0)	<0.001
	2 to <3 drink/day	190 (17.7)	149 (13.2)	203 (13.5)	41 (7.5)	<0.001
	<2 drinks/day	455 (42.4)	546 (48.4)	723 (47.9)	250 (46.0)	0.061
	Ex-drinking	58 (5.4)	47 (4.2)	96 (6.4)	40 (7.4)	0.035
	Non-drinking	256 (23.9)	307 (27.2)	425 (28.2)	186 (34.2)	<0.001
Regular exercise		113 (10.5)	141 (12.5)	310 (20.6)	113 (20.8)	<0.001
Use of antihypertensives		188 (17.5)	210 (18.6)	301 (20.0)	92 (16.9)	0.591
Women						
Number of participants		1370	2624	4257	1617	
Age (years)		56.3 (8.2)	56.0 (8.0)	58.4 (7.5)	59.0 (7.5)	<0.001
SBP (mmHg)		124.9 (19.9)	121.8 (18.9)	123.5 (19.4)	123.3 (19.1)	0.135
HbA1c (%)		5.05 (0.65)	5.06 (0.63)	5.09 (0.63)	5.09 (0.53)	0.026
TC (mg/dL)		202.2 (32.6)	204.7 (32.8)	208.9 (32.3)	208.4 (30.8)	<0.001
HDLC (mg/dL)		60.1 (14.2)	61.9 (14.4)	62.2 (14.2)	63.1 (14.5)	<0.001
BMI	<18.5 kg/m^2^	52 (3.8)	72 (2.7)	129 (3.0)	41 (2.5)	0.343
	18.5 to <25 kg/m^2^	779 (56.9)	1614 (61.5)	2682 (63.0)	1025 (63.4)	0.008
	25 to <30 kg/m^2^	438 (32.0)	785 (29.9)	1251 (29.4)	486 (30.1)	0.465
	≥30 kg/m^2^	101 (7.4)	153 (5.8)	195 (4.6)	65 (4.0)	<0.001
Smoking status	Current smoking	79 (5.8)	92 (3.5)	86 (2.0)	37 (2.3)	<0.001
	Ex-smoking	32 (2.3)	46 (1.8)	53 (1.2)	16 (1.0)	<0.001
	Non-smoking	1259 (91.9)	2486 (94.7)	4118 (96.7)	1564 (96.7)	<0.001
Alcohol drinking status	≥2 drinks/day	59 (4.3)	64 (2.4)	57 (1.3)	12 (0.7)	<0.001
	<2 drinks/day	200 (14.6)	398 (15.2)	513 (12.1)	163 (10.1)	<0.001
	Ex-drinking	32 (2.3)	38 (1.4)	54 (1.3)	31 (1.9)	0.284
	Non-drinking	1119 (81.7)	2171 (82.7)	3679 (86.4)	1421 (87.9)	<0.001
Regular exercise		135 (9.9)	266 (10.1)	525 (12.3)	215 (13.3)	<0.001
Use of antihypertensives		268 (19.6)	444 (16.9)	771 (18.1)	311 (19.2)	0.728
Postmenopausal state		977 (71.3)	1872 (71.3)	3439 (80.8)	1326 (82.0)	<0.001

Continuous variables are expressed as means (standard deviations), and categorical variables are expressed as numbers (percentages). *p* for trend was estimated by one-way analysis of variance for continuous variables and by chi-square test for categorical variables. Abbreviations: SBP, systolic blood pressure; HbA1c, glycated haemoglobin; TC, total cholesterol; HDLC, high-density lipoprotein cholesterol; BMI, body mass index.

**Table 2:** Food intake frequency according to milk intake frequency categories by sex, 2002–2005, Iwate-KENCO study.

Milk Intake Frequency	<2 Cups/Week	2 to <7 Cups/Week	7 to <12 Cups/Week	≥12 Cups/Week	*p* for Trend
Men					
Number of participants	1072	1129	1508	544	
Starchy foods (portion/week)	29.8 (11.3)	30.0 (11.0)	29.0 (10.6)	30.1 (12.0)	0.443
Fish (times/week)	9.3 (6.8)	10.1 (6.6)	10.5 (7.1)	11.2 (8.2)	<0.001
Soy products (times/week)	7.6 (4.5)	7.9 (4.1)	9.2 (4.7)	9.8 (5.0)	<0.001
Meat (times/week)	8.3 (2.5)	8.7 (2.5)	8.6 (2.8)	9.1 (3.5)	<0.001
Protein foods, (fish + soy)/meats ratio	2.1 (1.2)	2.1 (1.2)	2.3 (1.2)	2.5 (1.4)	<0.001
Vegetable (times/week)	26.2 (14.3)	27.3 (14.2)	30.7 (15.4)	32.0 (17.1)	<0.001
Fruit (times/week)	4.0 (4.5)	5.2 (4.9)	6.8 (5.8)	7.4 (6.4)	<0.001
Miso soup (cups/day)	2.5 (1.3)	2.5 (1.2)	2.5 (1.1)	2.5 (1.2)	0.870
Sugary drink (drinks/week)	3.0 (4.7)	2.7 (4.2)	2.6 (4.4)	3.1 (4.7)	0.487
Women					
Number of participants	1370	2624	4257	1617	
Starchy foods (portion/week)	25.2 (8.1)	25.7 (8.0)	25.2 (7.9)	26.2 (8.6)	0.042
Fish (times/week)	9.1 (7.0)	10.3 (7.0)	10.7 (7.1)	12.3 (8.6)	<0.001
Soy products (times/week)	8.2 (4.9)	8.9 (4.6)	9.7 (4.7)	10.7 (5.2)	<0.001
Meat (times/week)	8.4 (2.5)	8.8 (2.6)	8.6 (2.6)	8.9 (3.1)	0.008
Protein foods, (fish + soy)/meats ratio	2.1 (1.4)	2.2 (1.2)	2.4 (1.4)	2.7 (2.1)	<0.001
Vegetable (times/week)	30.2 (16.1)	32.4 (15.4)	35.2 (16.7)	38.2 (18.1)	<0.001
Fruit (times/week)	5.9 (5.7)	7.2 (5.9)	8.6 (6.3)	9.8 (7.2)	<0.001
Miso soup (cups/day)	2.1 (1.0)	2.2 (1.0)	2.1 (1.0)	2.2 (1.0)	0.399
Sugary drink (drinks/week)	1.6 (3.4)	1.5 (2.8)	1.4 (3.0)	1.6 (3.4)	0.648

Data are expressed as mean (standard deviation). *p* for trend was estimated using one-way analysis of variance.

**Table 3:** Multivariate adjusted hazard ratios for total stroke, ischemic stroke, and hamorrhagic stroke, according to milk intake frequency categories by sex, 2002–2014, Iwate-KENCO study.

Milk Intake Frequency	<2 Cups/Week	2 to <7 Cups/Week	7 to <12 Cups/Week	≥12 Cups/Week	*p* for Trend
Men					
Person-years	11,268	11,848	15,986	5764	
Total stroke					
Number of cases	49	64	70	25	
Crude incidence rate	4.35	5.40	4.38	4.34	
HR (95% CI)					
Model 1	1	1.18 (0.81–1.71)	0.84 (0.58–1.21)	0.83 (0.51–1.35)	0.500
Model 2	1	1.22 (0.84–1.77)	0.92 (0.63–1.33)	0.93 (0.57–1.51)	0.885
Model 3	1	1.24 (0.84–1.82)	0.93 (0.63–1.36)	0.95 (0.57–1.57)	0.985
Model 4	1	1.21 (0.83–1.79)	0.99 (0.67–1.46)	0.97 (0.59–1.61)	0.810
Ischemic stroke					
Number of cases	31	42	50	11	
Crude incidence rate	2.75	3.54	3.13	1.91	
HR (95% CI)					
Model 1	1	1.21 (0.76–1.93)	0.92 (0.58–1.44)	0.56 (0.28–1.12)	0.165
Model 2	1	1.24 (0.78–1.98)	0.99 (0.62–1.56)	0.61 (0.31–1.23)	0.289
Model 3	1	1.28 (0.80–2.06)	1.01 (0.63–1.62)	0.61 (0.30–1.27)	0.364
Model 4	1	1.27 (0.79–2.05)	1.07 (0.66–1.71)	0.64 (0.31–1.34)	0.491
Haemorrhagic stroke					
Number of cases	18	21	20	13	
Crude incidence rate	1.60	1.77	1.25	2.26	
HR (95% CI)					
Model 1	1	1.06 (0.57–2.00)	0.68 (0.36–1.29)	1.23 (0.60–2.53)	0.586
Model 2	1	1.12 (0.59–2.11)	0.77 (0.40–1.47)	1.42 (0.69–2.95)	0.331
Model 3	1	1.09 (0.56–2.10)	0.76 (0.39–1.49)	1.44 (0.68–3.04)	0.295
Model 4	1	1.04 (0.53–2.01)	0.82 (0.42–1.61)	1.39 (0.66–2.94)	0.281
Women					
Person-years	14,657	28,098	45,822	17,218	
Total stroke					
Number of cases	50	61	104	55	
Crude incidence rate	3.41	2.17	2.27	3.19	
HR (95% CI)					
Model 1	1	0.65 (0.45–0.95)	0.59 (0.42–0.83)	0.80 (0.55–1.18)	0.536
Model 2	1	0.66 (0.45–0.96)	0.60 (0.43–0.84)	0.81 (0.55–1.20)	0.590
Model 3	1	0.66 (0.44–0.98)	0.67 (0.47–0.95)	0.91 (0.61–1.37)	0.909
Model 4	1	0.71 (0.47–1.05)	0.73 (0.51–1.05	1.03 (0.68–1.55)	0.567
Ischemic stroke					
Number of cases	28	31	42	28	
Crude incidence rate	1.91	1.10	0.92	1.63	
HR (95% CI)					
Model 1	1	0.60 (0.36–0.999)	0.41 (0.26–0.67)	0.70 (0.41–1.18)	0.153
Model 2	1	0.60 (0.36–1.01)	0.41 (0.26–0.67)	0.70 (0.41–1.18)	0.152
Model 3	1	0.65 (0.38–1.11)	0.48 (0.29–0.79)	0.77 (0.44–1.35)	0.322
Model 4	1	0.69 (0.40–1.18)	0.53 (0.32–0.88)	0.89 (0.50–1.57)	0.572
Haemorrhagic stroke					
Number of cases	22	29	61	26	
Crude incidence rate	1.50	1.03	1.33	1.51	
HR (95% CI)					
Model 1	1	0.70 (0.40–1.22)	0.81 (0.50–1.33)	0.90 (0.51–1.59)	0.664
Model 2	1	0.71 (0.41–1.24)	0.84 (0.51–1.37)	0.93 (0.53–1.65)	0.584
Model 3	1	0.65 (0.36–1.17)	0.91 (0.55–1.53)	1.06 (0.58–1.93)	0.300
Model 4	1	0.70 (0.39–1.26)	0.98 (0.59–1.64)	1.17 (0.64–2.15)	0.195

Crude incidence rate is expressed as number of cases per 1000 person-years. Model 1: adjusted for age. Model 2: Model 1 + smoking status, alcohol consumption status, and exercise habits. Model 3: Model 2 + fruits and vegetables intake frequency and the ratio of total fish and soy products consumption to meat consumption. Model 4: Model 3 + body mass index, systolic blood pressure, glycated haemoglobin, total cholesterol, high-density lipoprotein cholesterol, use of antihypertensives, and menopausal state (if women). Abbreviations: HR, hazard ratio; CI, confidence interval.

## Data Availability

No additional data are available.
